# Human menstrual blood-derived stromal/stem cells modulate functional features of natural killer cells

**DOI:** 10.1038/s41598-019-46316-3

**Published:** 2019-07-10

**Authors:** Mohammad-Reza Shokri, Mahmood Bozorgmehr, Alireza Ghanavatinejad, Reza Falak, Mehdi Aleahmad, Somaieh Kazemnejad, Fazel Shokri, Amir-Hassan Zarnani

**Affiliations:** 1 Department of Immunology, School of Medicine, Iran University of Medical Sciences, Tehran, Iran; 2 Department of Immunology, School of Public Health, Tehran University of Medical Sciences, Tehran, Iran; 3 Oncopathology Research Center, Iran University of Medical Sciences, Tehran, Iran; 4 Reproductive Immunology Research Center, Avicenna Research Institute, ACECR, Tehran, Iran; 5 Reproductive Biotechnology Research Center, Avicenna Research Institute, ACECR, Tehran, Iran; 6 Immunology Research Center, Iran University of Medical Sciences, Tehran, Iran

**Keywords:** NK cells, Mesenchymal stem cells

## Abstract

Although natural killer (NK) cells play a crucial role in the maintenance of a successful pregnancy, their cytotoxic activity should be tightly controlled. We hypothesized that endometrial mesenchymal stromal/stem cells (eMSCs) could potentially attenuate the functional features of NK cells. Herein, we assessed immunomodulatory effects of menstrual blood-derived stromal/stem cells (MenSCs), as a surrogate for eMSCs, on NK cells function. Our results showed that MenSCs induced proliferation of NK cells. However, IFN-γ/IL-1β pretreated MenSCs significantly inhibited NK cell proliferation. Of 41 growth factors tested, MenSCs produced lower levels of insulin-like growth factor binding proteins (IGFBPs) 1–4, VEGF-A, β-NGF, and M-CSF compared to bone marrow-derived mesenchymal stem cells (BMSCs). MenSCs displayed high activity of IDO upon IFN-γ treatment. The antiproliferative potential of IFN-γ/IL-1β-pretreated MenSCs was mediated through IL-6 and TGF-β. MenSCs impaired the cytotoxic activity of NK cells on K562 cells, consistent with the lower expression of perforin, granzymes A, and B. We also observed that *in vitro* decidualization of MenSCs in the presence of IFN-γ reduced the inhibitory effect of MenSCs on NK cell cytotoxicity against K562 target cells. Additionally, MenSCs were found to be prone to NK cell-mediated lysis in an MHC-independent manner. Our findings imply that dysregulation of NK cells in such pregnancy-related disorders as miscarriage may stem from dysfunctioning of eMSCs.

## Introduction

Natural killer (NK) cells are the main component of the endometrial innate immune system, playing a crucial role in maintaining a successful pregnancy^1^. Decidual NK (dNK) cells are the most abundant immune cells in the pregnant endometrium, comprising 50–70% of the total lymphoid cells^2^. There currently is not a consensus on the origin of dNK cells. Reportedly, they could either be differentiated *in situ* from CD34+ precursors or derived from peripheral blood NK cells recruited into the endometrium during pregnancy^1,3–5^. There is a preferential active recruitment of NK cells into the endometrium at the onset of pregnancy mediated by endometrial stromal cells- and trophoblast-derived chemokines, which exert pregnancy-friendly regulatory functions^6^.

Among the well-known functions attributed to the dNK cells, regulation of tissue homeostasis and vascular remodeling are of utmost importance^7,8^. dNK cells are mostly of regulatory subtype and differ fundamentally with their peripheral blood cytotoxic counterpart regarding the phenotype, function, and gene expression^9^. Several mechanisms have been proposed for hypocytotoxicity of dNK cells including the interaction of their inhibitory receptors with non-classical HLA^10^ and lower expression of CD16^10,11^. Despite several immunomodulatory mechanisms proposed so far for the regulation of endometrial immune cells including dNK cells, investigations to explore new mechanisms are actively ongoing. One main emerging mechanism is the immunomodulation exerted by mesenchymal stem cells (MSCs). In this context, various studies have reported negative functional modulation of B, T, NK, monocytes, and dendritic cells, as well as induction of regulatory T cells by MSCs derived from various sources through different mechanisms including cell-cell contact or secreted soluble factors^12–19^.

Menstrual blood stromal/stem cells (MenSCs), originated from the endometrial layer of the uterus, are a type of newly introduced MSC with considerable multi-lineage differentiation capacity^20–26^. In spite of several reports on the immunomodulatory impact of MSCs from such sources as bone marrow^14–17^ and adipose tissue^27–29^, there is a paucity of reports concerning MenSCs impacts on immune cells and responses. We recently showed that, depending on their concentration, MenSCs could either inhibit or stimulate an allogeneic mixed lymphocyte reaction (MLR) and also inhibit optimal maturation of monocyte-derived dendritic cells^30,31^. However, the probable immunomodulatory impact of MenSCs on NK cells awaits further investigations. Here, we hypothesized that MenSCs, as a surrogate for endometrial stromal/stem cells (eMSCs), could potentially attenuate functional features of NK cells and reprogram these cells toward cells with dNK phenotype and activity.

## Results

### MenSCs exhibit multi-lineage differentiation capacity and present dual expression of mesenchymal and embryonic cell markers

**Figure 1:**
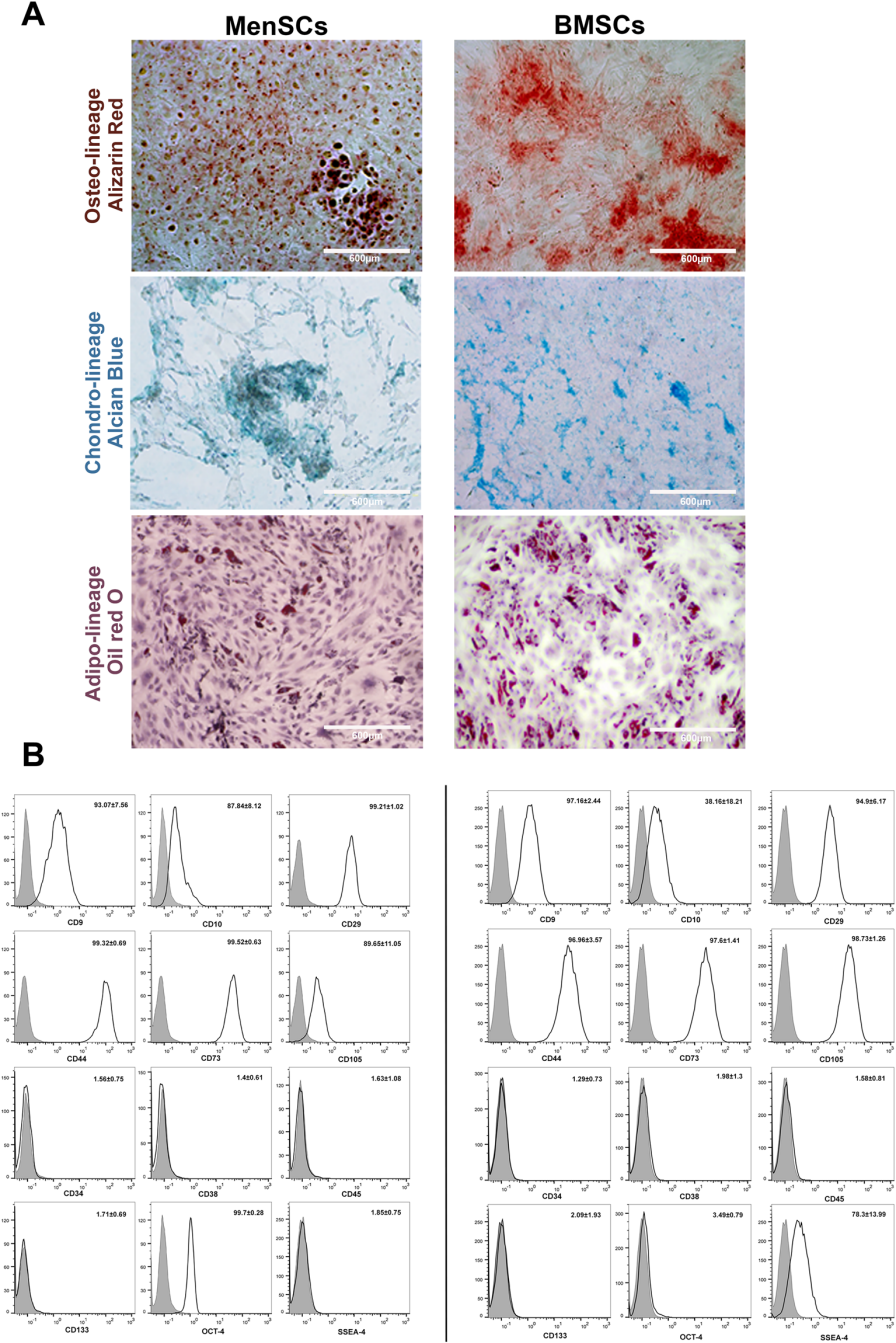
Immunophenotype and multi-lineage differentiation potential of MenSCs and BMSCs. (**A**) MSC differentiation into osteoblasts, chondrocytes, and adipocytes was assessed using Alizarin Red, Alcian Blue, and Oil Red O staining, respectively. (**B**) Immunophenotyping of MenSCs and BMSCs. Gray histograms correspond to isotype control. Data are expressed as mean ± SD and are representative of 4 MenSCs and BMSCs donors. Images were photographed by an Olympus microscope (BX-51) equipped with a CCD camera (DP71) camera.

Both MenSCs and bone marrow-derived mesenchymal stem cells (BMSCs) were capable of differentiating into adipogenic, chondrogenic, and osteogenic lineages. However, MenSCs showed less potency to differentiate into osteogenic and adipogenic lineages compared to BMSCs (Fig. 1A). MenSCs and BMSCs expressed MSCs markers including CD9, CD10, CD29, CD44, CD73, and CD105, but failed to express CD34, CD38, CD45 and, CD133. MenSCs also expressed Oct-4, but were found to be negative for SSEA-4, while the opposite pattern was observed for BMSCs (Fig. 1B).

### MenSCs support the proliferation of NK cells in a dose-dependent and paracrine manner

**Figure 2:**
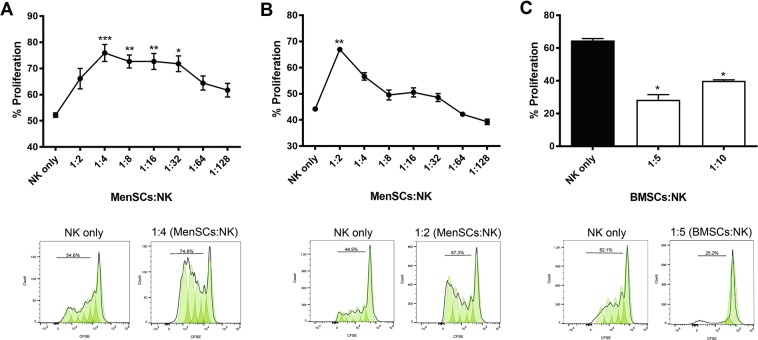
Effect of MenSCs and BMSCs on the proliferation of NK cells. Freshly isolated NK cells were co-cultured (**A**) directly or in a (**B**) transwell system at different ratios with mitomycin C-pretreated MenSCs in the presence of 100 U/mL of IL-2 for 5 days. The proliferation of NK cells was evaluated using CFSE flow cytometry. BMSCs co-cultured with NK cells served as a control. (**C**) The data represent the mean ± SEM of 8 MenSCs and 4 BMSCs from different donors. P values less than 0.05, 0.01 and 0.001 are shown with *, **, and ***, respectively.

BMSCs have been shown to suppress NK cell proliferation markedly^15^. To assess whether MenSCs could exert a similar inhibitory effect on NK cells, mitomycin C-pretreated MenSCs were co-cultured with CFSE-labeled NK cells in the presence of IL-2. As shown in Fig. 2A, MenSCs affected proliferation of NK cells in a dose-dependent manner with a maximum supportive impact at a ratio of 1:4 (MenSCs:NK) (P < 0.001). By increasing MenSC:NK cell ratios, the proliferation of NK cells was decreased. To determine whether the supportive effect of MenSCs on NK cell proliferation is mediated by cell-cell contact or soluble factors, secreted by MenSCs, co-culturing in a transwell system was employed. MenSCs secretome significantly increased proliferation of NK cells at a MenSC:NK cell ratio of 1:2 (P < 0.01). Subsequently, NK cell proliferation was reduced and reached the level observed in control wells (Fig. 2B). In parallel and as a gold-standard cell type, the effect of IFN-γ-pretreated BMSCs on the proliferation of NK cells was also investigated. As shown in Fig. 1C, and in line with previous studies, BMSCs strongly suppressed NK cell proliferation at 1:5 and 1:10 ratios (BMSCs:NK) (P < 0.05).

### MenSCs secretome contain fewer IGFBPs compared to BMSCs

**Figure 3:**
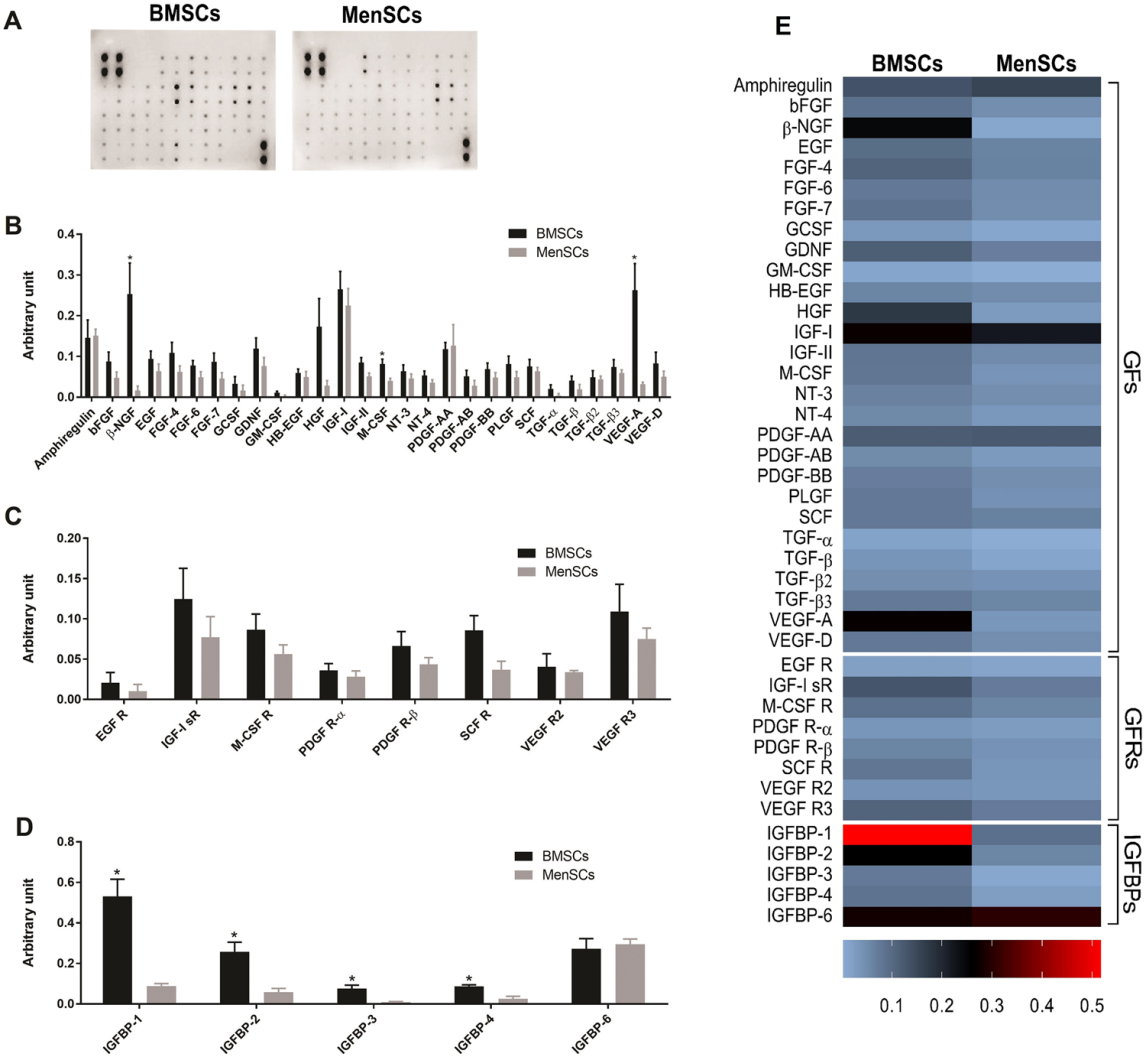
Analysis of MenSCs and BMSCs secretome by growth factor membrane antibody array. (**A**,**B**) Relative amounts of 41 growth factors (GFs), (**C**) growth factor receptors (GFRs), and (**D**) insulin-like growth factor binding proteins (IGFBPs) were assessed in culture supernatants of 4 MenSCs and BMSCs from different donors. Part A outlines full-length blot without any manipulation including cropping. Blots of BMSCs and MenSCs were developed with the same procedure and exposure times. P values less than 0.05 are shown with *. (**E**) Heat map of the expressed proteins is shown.

To determine the mechanisms underlying NK cell proliferation following co-culturing with MenSCs, the secretome of MenSCs and BMSCs were tested for relative concentrations of 41 different growth factors, their receptors, and binding proteins using membrane-based antibody array (Fig. 3A). We found that except for β-NGF and VEGF-A which were produced in significantly higher amounts by BMSCs (P < 0.05), the secretome of both MSCs contained comparable amounts of other growth factors tested (Fig. 3B). No significant difference was found for soluble growth factor receptors (Fig. 3C). Remarkably, the levels of IGFBP-1, IGFBP-2, IGFBP-3, and IGFBP-4 were found to be significantly higher in BMSCs compared to MenSCs (P < 0.05) (Fig. 3D,E).

### IFN-γ/IL-1β-pretreated MenSCs suppress NK cell proliferation

**Figure 4:**
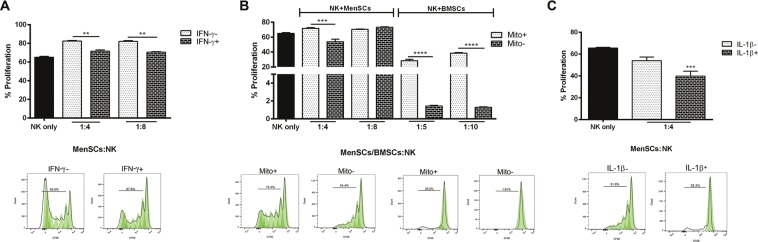
Effect of pro-inflammatory cytokines and mitomycin C pretreatment of MenSCs on NK cells proliferation. (**A**) Mitomycin C-inactivated MenSCs were co-cultured with IL-2-activated NK cells in the presence or absence of IFN-γ. (**B**) In parallel, the effect of mitomycin C inactivation of IFN-γ pretreated MenSCs and BMSCs was assessed on NK cell proliferation. (**C**) IFN-γ pretreated MenSCs were also co-cultured with NK cells in the presence or absence of IL-1β. The proliferation of CFSE-labeled NK cells was measured after 5 days. Results are represented as mean ± SEM of 6 MenSCs and 4 BMSCs from different donors. P values less than 0.01, 0.001, and 0.0001 were shown with **, ***, and ****, respectively.

To evaluate whether IFN-γ could revert supportive activity of MenSCs on NK cell proliferation, MenSCs were pretreated with IFN-γ for 48 hrs before co-culture with NK cells. Although IFN-γ pretreatment of MenSCs significantly reduced their supportive effect on NK cell proliferation (P < 0.01), the NK proliferation level was still not inhibited enough compared to the control (Fig. 4A). In the next step, we tested whether mitomycin C pretreatment of MenSCs may have a major effect on the proliferation of NK cells. The results showed that mitomycin C pretreatment of both MSC types reduced their capacity to suppress NK cell proliferation (P < 0.001 for MenSCs; P < 0.0001 for BMSCs) (Fig. 4B). All subsequent tests were therefore performed on IFN-γ-pretreated MSCs without inactivation with mitomycin C. We then tested whether the simultaneous pretreatment of MenSCs with two pro-inflammatory cytokines, IFN-γ and IL-1β, could induce immunosuppressive phenotype in these cells. Interestingly, our results showed that MenSCs pretreated with these cytokines significantly suppressed NK cell proliferation (P < 0.001) (Fig. 4C).

### IFN-γ but not IL-1β induce IDO activity in MenSCs

**Figure 5:**
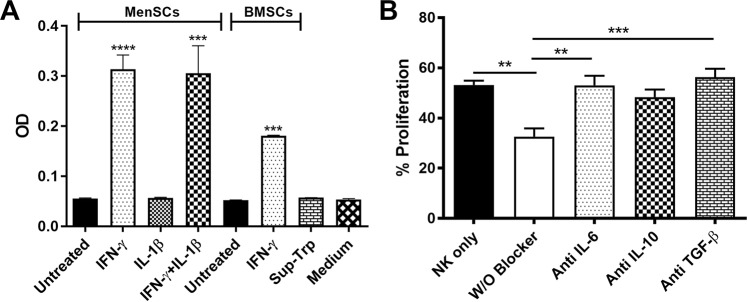
Effect of cytokines on MenSC-mediated inhibition of NK-cell proliferation. (**A**) Effect of treatment with pro-inflammatory cytokines (IFN-γ and/or IL-1β) on IDO activity in the supernatants of MenSCs was assessed. BMSCs stimulated with IFN-γ served as the positive control. Negative controls included untreated cells, supernatants without tryptophan addition (Sup-Trp) and medium alone. (**B**) CFSE-labeled NK cells were co-cultured with IFN-γ/IL-1β pretreated MenSCs in the presence of IL-2 and neutralizing antibodies against IL-6, IL-10, and TGF-β for 5 days and proliferation were measured by flow cytometry. Results are represented as mean ± SEM of 6 MenSCs and 4 BMSCs from different donors. P values less than 0.01, 0.001 and, 0.0001 are shown with **, *** and, ****, respectively.

IDO activity is induced upon stimulation with pro-inflammatory cytokines, IFN-γ, TNF-α, or IL-1β^32^. In this regard, MenSCs were treated with IFN-γ and/or IL-1β, and the level of kynurenine was measured in the cell culture supernatant. We observed that IFN-γ induced IDO activity in MenSCs (P < 0.0001), but it was not the case for IL-1β. Simultaneous treatment of MenSCs with IFN-γ and IL-1β did not significantly enhance IDO activity in MenSCs compared to IFN-γ treatment alone. As expected, IFN-γ treatment of BMSCs caused a significant induction of IDO activity (P < 0.001) (Fig. 5A).

### IL-6 and TGF-β mediate antiproliferative potential of IFN-γ/IL-1β-pretreated MenSCs

We then tested the potential role of IL-6, IL-10, and TGF-β in MenSCs-mediated inhibition of NK cell proliferation using neutralizing antibodies. Blocking of IL-6 (P < 0.01) or TGF-β (P < 0.001) almost completely restored the proliferation of NK cells co-cultured with MenSCs, whereas IL-10 blockade did not exert any statistically significant effect (Fig. 5B).

### IFN-γ/β-pretreated MenSCs profoundly inhibit NK cell cytotoxicity through down-regulation of perforin and granzymes

**Figure 6:**
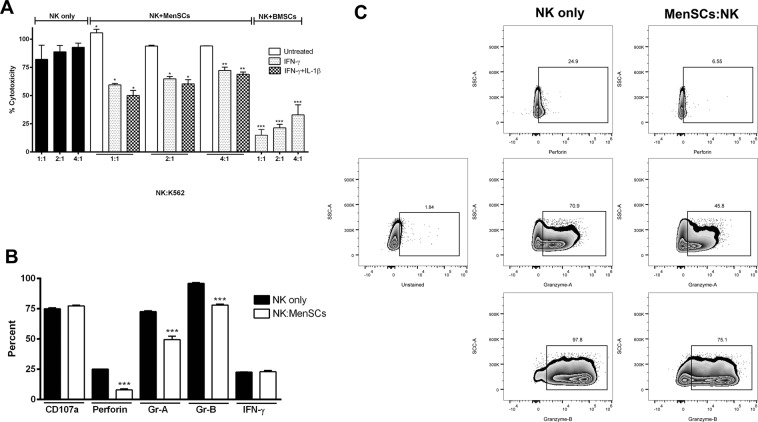
Effect of MenSCs on NK cell cytotoxicity. (**A**) IL-2-activated NK cells were cultured with IFN-γ/IL-1β-activated MenSCs for 5 days. Cytotoxicity of NK cells on K562 cells at different ratios was then assessed by cAM fluorimetric assay. IFN-γ-pretreated BMSCs served as positive control. (**B**,**C**) Effect of MenSCs co-culturing on the expression of cytotoxic markers was evaluated by flow cytometry. Results are represented as mean ± SEM of 6 MenSCs and 4 BMSCs from different donors. P values less than 0.05, 0.01, and 0.001 are shown with *, ** and, ***, respectively.

In the next step, we tested whether IFN-γ/IL-1β pretreated MenSCs could functionally hamper NK cell cytotoxicity against K562 target cells. We observed that contrary to its proliferative effects, IFN-γ pretreatment alone was able to significantly suppress NK cell cytotoxicity at all tested ratios (P < 0.05-0.0.01). Although NK cells co-cultured with IFN-γ/IL-1β-pretreated MenSCs showed lower cytotoxicity compared to those co-cultured with IFN-γ-pretreated MenSCs, this did not reach a statistically significant level. BMSCs, as a positive control, considerably halted cytotoxicity of NK cells at all co-culture ratios (P < 0.001). As with proliferation assay, MenSCs without IFN-γ pretreatment, however, exerted opposite effect and up-regulated cytotoxicity of NK cells against K562 cells (P < 0.05) (Fig. 6A). To determine the mechanisms responsible for impaired NK cell cytotoxicity following co-culturing with MenSCs, the levels of NK cells cytotoxic markers were measured. We found that co-culture with IFN-γ/IL-1β-pretreated MenSCs profoundly reduced the levels of perforin, granzymes A, and B in NK cells (P < 0.001) (Fig. 6B,C). MenSCs did not affect the levels of CD107a and IFN-γ expression in NK cells.

**Figure 7:**
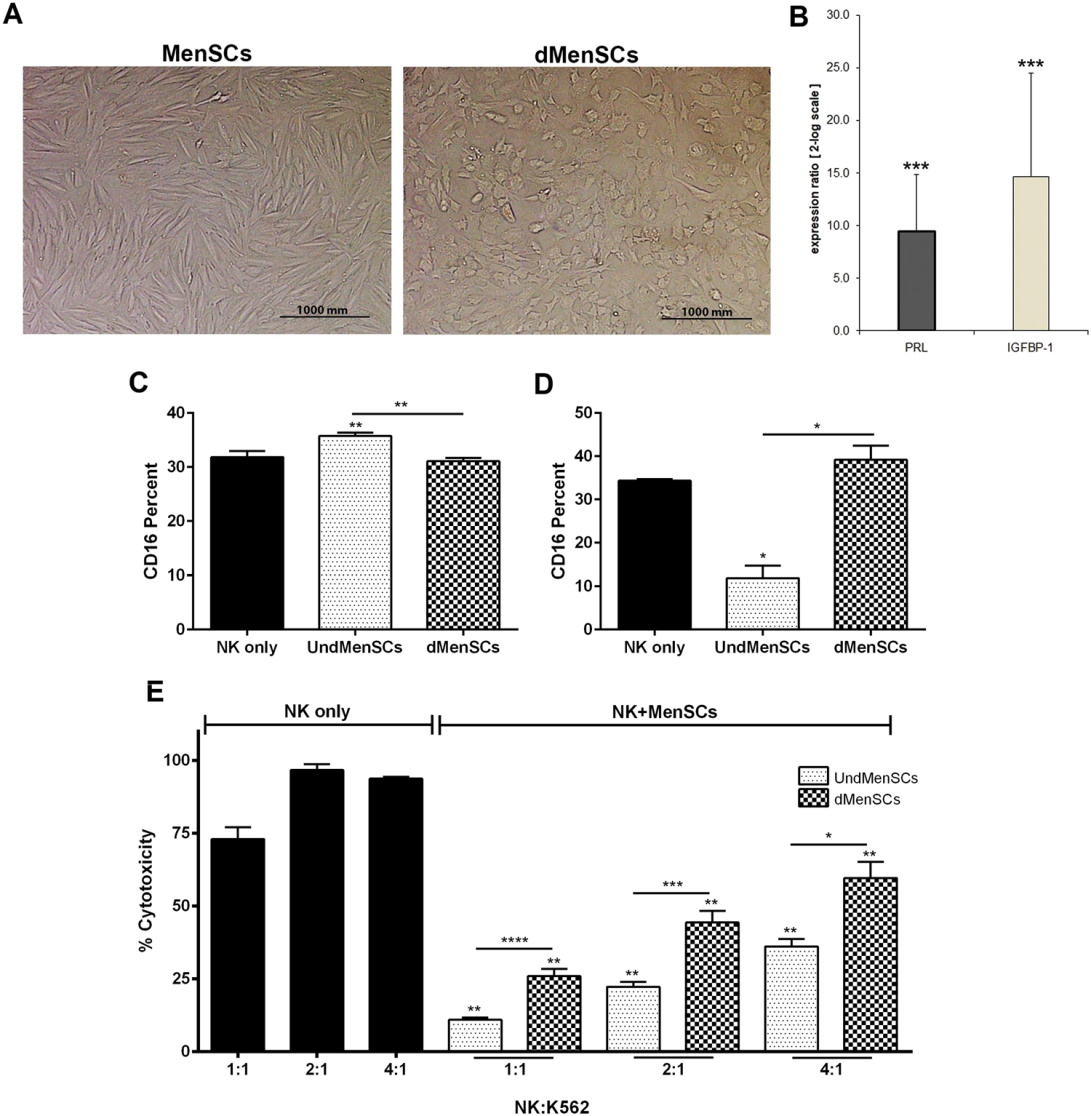
Effect of MenSCs decidualization on cytotoxicity of NK cells. (**A**) MenSCs were *in vitro* decidualized in the presence or absence of IFN-γ, and (**B**) the expression of *PRL* and *IGFBP*-1 was assessed by real-time RT-PCR in comparison to control cells. (**C**) Effect of MenSCs decidualized in the absence of IFN-γ on CD16 expression in NK cells was investigated after 2 days co-culture by flow cytometry. dMenSCs significantly (P < 0.01) reduced percent of CD16 expression in CD56 + NK cells. (dMenSCs: 31.06% ± 1.38, UndMenSCs: 35.73% ± 1.35 and NK only: 31.85% ± 1.95) (**D**) MenSCs decidualized in the presence of IFN-γ had no significant effect on CD16 expression in comparison to NK cells cultured alone (NK only), but (**E**) significantly reduced NK cytotoxicity against K562 cells at all ratios. P values less than 0.05, 0.01, 0.001, and 0.0001 are shown with *, **, *** and, **** respectively. Images were photographed by an Olympus microscope (BX-51) equipped with a CCD camera (DP71) camera. UndMenSCs: Undifferentiated MenSCs, dMenSCs: decidulized MenSCs.

We also observed that decidualized (dMenSCs) (Fig. 7A), expressing high levels of *PRL* and *IGFBP-1* (Fig. 7B). Next, we tested whether decidualization could potentially exert modulatory effect on NK cell CD16 expression and cytotoxicity. We performed *in vitro* decidualization in either the presence or absence of IFN-γ. Compared to control NK cells cultured in the absence of MenSCs, decidualization did not affect CD16 expression in both experiments. However, undecidualized MenSCs significantly reduced CD16 expression compared to NK cells cultured alone or co-cultured with decidualized MenSCs only when they were stimulated with IFN-γ (Fig. 7C,D). Regarding cytotoxicity, IFN-γ pretreatment in both decidualized and undecidualized MenSCs resulted in a significant decrease of cytotoxicity of NK cells against K562 cells at all ratios (P < 0.01). Surprisingly, however, the extent of cytotoxicity inhibition was higher in NK cells co-cultured with undecidualized compared to decidualized MenSCs (P < 0.05–0.0001) (Fig. 7E).

### NK cells kill MenSCs in a time-dependent and MHC-independent manner

**Figure 8:**
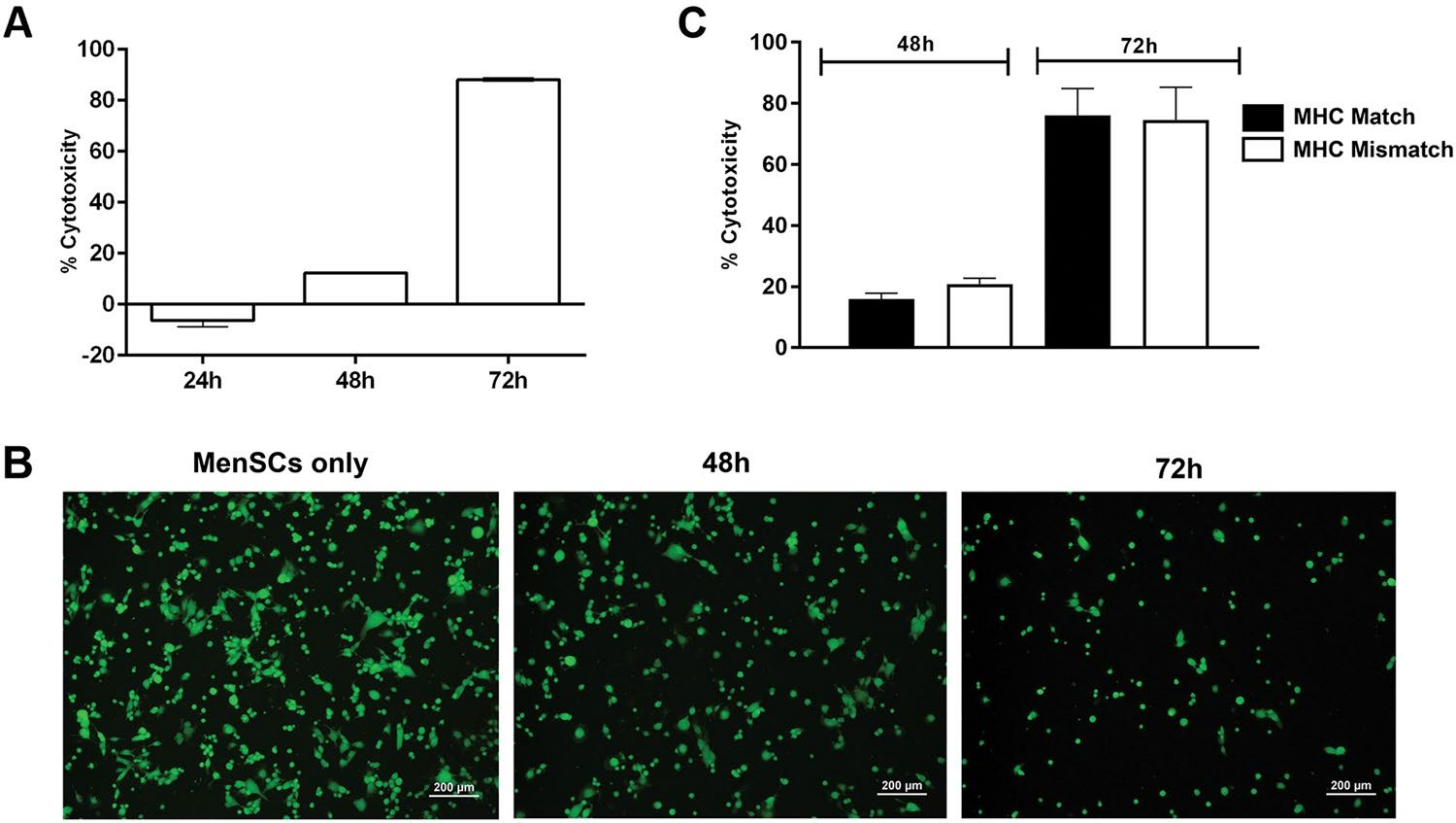
NK cell-mediated MenSCs lysis. (**A**) NK cells were co-cultured with allogeneic cAM-labeled MenSCs from 6 donors in the presence of IL-2, and the level of cytotoxicity was measured at different time points by fluorimetry. (**B**) Viable cells were monitored under a fluorescent microscope at different time points. (**C**) IL-2-activated NK cells from 3 different female donors were cultured with 3 allogeneic (MHC Mismatch) or syngeneic (MHC Match) MenSCs. The level of cytotoxicity was then measured as above. Images were photographed by an Olympus microscope (BX-51) equipped with a CCD camera (DP71) camera.

In co-culture systems containing NK cells and allogeneic MenSCs, we observed that the number of MenSCs progressively decreased during the cell culture period. Thus, we tested whether NK cells can kill MenSCs. MenSCs, as target cells, were co-cultured with NK cells and the level of cytotoxicity was tested by cAM fluorimetric assay at different time points. NK cells exerted cytotoxic effects on allogeneic MenSCs which reached a statistically significant level from 48 hrs upward (P < 0.0001) (Fig. 8A,B). To realize whether NK cytotoxicity against MenSCs is due to MHC disparity, cytotoxicity experiment was repeated with MHC-matched MenSCs. The results of this experiment clearly showed that akin to MHC-mismatched MenSCs, NK cells killed autologous MenSCs (Fig. 8C).

## Discussion

Although NK cells are major players at the feto-maternal interface for induction of tolerance to the semi-allogeneic fetus, the mechanisms responsible for their regulation are still not fully understood. Here, we showed that upon co-culturing of pro-inflammatory cytokines-pretreated MenSCs with peripheral blood NK cells, these cells displayed a significantly reduced cell proliferation, an impaired cytotoxic activity, along with a reduced perforin and granzymes production, a “pregnancy-friendly phenotype.” Although the contribution of several uterine microenvironment moieties including decidual CD14 + cells^33^, trophoblast cells, pregnancy hormones, and cytokines^34^ in modulation of NK cell function has already been reported, this is for the first time that immunomodulatory role of eMSCs -the most abundant endometrial cells- on NK cells is investigated. MenSCs are originated mainly from endometrial functional layer and to a lesser extent from the endometrial basal layer and share similar characteristics with eMSCs. Based on these features, lack of ethical issue and the non-invasive method of collection; we used MenSCs as a surrogate for eMSCs.

Our results showed that unlike BMSCs, which halted NK cell proliferation, MenSCs enhanced proliferation of NK cells. This finding was also in contrary to the inhibitory action of stem cells derived from other sources on NK cell proliferation^35,36^. An ascending pattern of NK cell proliferation from 1:2 to 1:4 (MenSCs:NK) ratios indicated a negative effect of MenSCs cell-cell contact on NK cell proliferation. However, the descending pattern of NK cell proliferation from 1:4 ratio (MenSCs:NK) upward prompted us to test the effect of soluble factors released from MenSCs on NK cell proliferation. The results clearly showed that soluble factors released from MenSCs, supported NK cell proliferation. We also observed that MenSCs conditioned media contained significantly lower amounts of IGFBP-1–4 compared to BMSCs which may cause availability of higher levels of bioactive IGF-1, a cytokine known to exert a positive effect on NK proliferation^37^. In line with our finding, a previous study showed that IGFBP-3 transcript is tended to be down-regulated in MenSCs among 768 genes differentially expressed by MenSCs and BMSCs^38^. Supportive effect of MenSCs on the proliferation of CD34 + CD133 + hematopoietic stem cells has also been reported earlier^39^. Indeed, stem cells derived from umbilical cord showed the same supportive action on umbilical cord blood NK cells^40^. MenSCs pretreated with both IFN-γ and IL-1β, however, significantly reduced the proliferation of NK cells. Luz-Crawford *et al*. also showed that MenSCs displayed a suppressive effect on proliferation of T cells when pretreated with both cytokines. Importantly, IFN-γ and IL-1β are the main pro-inflammatory cytokines present in the decidualized endometrium. They may, therefore, be involved in controlling dNK cell proliferation through modulatory activity on eMSCs. In line with such assumption, Croxatto *et al*. showed that MSCs derived from decidua tissue strongly inhibited IL-15-stimulated NK cell proliferation^35^. Blocking assay showed IL-6 and TGF-β were involved in the antiproliferative effect of pro-inflammatory cytokine-treated MenSCs. These cytokines, along with prostaglandin E2 (PGE2), indoleamine 2,3 dioxygenase (IDO), and programmed cell death-ligand 1 (PD-L1) have been implicated in the suppressive effect of BMSCs on the proliferation of NK cells^41–45^. We observed that IFN-γ but not IL-1β induced IDO activity in MenSCs. Based on the finding that IFN-γ-pretreated MenSCs were unable to inhibit NK cell proliferation, it can be concluded that other factors, mentioned above, are more likely to be responsible for the induction of NK cell antiproliferative potency in MenSCs following IFN-γ/IL-1β pretreatment.

MenSCs pretreated with IFN-γ or IFN-γ plus IL-1β remarkably inhibited NK cell cytotoxicity against K562 cells. Contrary to the proliferation assay, IL-1β did not synergistically act with IFN-γ indicating that different functional features of NK cells are independently regulated. IL-2-activated NK cells show increased expression of such activating receptors as NKp30, NKG2D, and also CD69 activation marker. Co-culturing with BMSCs, however, caused expression of these markers to be normalized^15,46^. Whether the inhibition of NK cell cytotoxicity following co-culturing with MenSCs is mediated through the same mechanism needs to be elucidated. However, we found that co-culturing with MenSCs reduced the expression of perforin and granzymes in NK cells supporting inhibition of NK cell cytotoxicity by MenSCs. We observed that NK cells co-cultured with MenSCs were able to kill MenSCs in an MHC-independent manner. Therefore, it is plausible that at least a part of the reduction in perforin, granzymes A, and B content of NK cells co-cultured with MenSCs is due to the release of these molecules in response to the MenSCs stimulation instead of being down-regulated at the gene or protein level. Our finding is in contrast with previous reports showing that reduced cytotoxicity of NK cells following co-culturing with BMSCs was not due to the diminished levels of granzymes and perforin^47,48^ suggesting that modulation of NK cell activity by different sources of stem cells may be mediated through different mechanisms.

Next, we tested the effect of decidualization of MenSCs on their modulatory effects on NK cell cytotoxicity. We observed that NK cells co-cultured with the MenSCs decidualized in the presence of IFN-γ, showed considerably reduced cytotoxicity compared to the NK cells cultured alone, but had higher cytotoxicity compared to the NK cells co-cultured with undecidualized MenSCs. This means that, in the presence of IFN-γ, decidualzation to somehow restore the cytotoxicity of NK cells, albeit not to the control levels. Decidualization exerted different effects on MenSCs capacity to alter NK cell cytotoxicity depending on presence or absence of IFN-γ during decidualization process suggesting that pro-inflammatory cytokines present in the endometrium during implantation period could affect immunomodulatory effects of decidualized endometrium. Collectively, these findings suggest that decidualization tunes the functional capacity of NK cells and keep their activity under control. Notably, reduced density of dNK cells has been linked to enhanced extravillous trophoblast invasion in invasive molar pregnancy and placenta accreta^49,50^. It has been evident that endometrial MSCs deficiency is linked to pregnancy failure through aberrant decidualization^51^. Now we can postulate that aberrant decidualization may trigger pregnancy loss through impaired endometrial control over NK cell immunomodulation.

While MenSCs moduleted NK cell activity, NK cells tended to kill MenSCs in a time-dependent manner. Susceptibility of BMSCs to NK cell-mediated lysis has been reported earlier^52^. Lysis of trophoblasts is a known function of dNK cells through which the degree of placental invasion is controlled^53,54^. In this context, NK cell-mediated lysis of eMSCs can be regarded as an essential regulatory mechanism for the establishment of endometrial homeostasis. More importantly, we observed that killing of MenSCs by pre-activated NK cells is not dependent upon MHC disparity, suggesting that this effect is a physiological rather than a pathological phenomenon. It is postulated that the cytotoxicity of NK cells against BMSCs is inversely correlated with IFN-γ activation of BMSCs^52^. However, our results showed that the degree of MenSCs lysis by NK cells was not affected by the activation state of MenSCs, again pointing to the intrinsic functional difference between two cell types.

Collectively, our results imply that MenSCs, as a surrogate for eMSCs, could be regarded as an important instructor of the local uterine immune system, exemplified here by their potential action on NK cells. These results suggest that eMSCs may contribute to the induction of tolerogenic microenvironment during early pregnancy. It could be hypothesized that impairment of the eMSCs function may lead to miscarriages mediated by immunological imbalance.

## Methods

### Donor selection

Menstrual blood samples were obtained from 18 apparently healthy volunteer women at ages ranged between 25 and 35 years based on the following inclusion and exclusion criteria. The recruited women had at least one live birth without a history of abortion. They also had negative test results for blood transmittable viruses (HIV, HBV, and HCV) and had no sign or symptoms of endometriosis and autoimmune diseases. Those with a history of vaginal infection, consumption of oral contraceptives, a nonsteroidal anti-inflammatory drug, and corticosteroids during the last 3 months were excluded. BMSCs from four age-matched healthy donors were provided by Avicenna Research Institute (ARI). All methods and experiments of this research study were approved by the ethics committees of Iran University of Medical Sciences (IUMS) and ARI and were performed in accordance with the relevant guidelines and regulations of IUMS and ARI. All participants signed a written informed consent prior to participating in the study.

### MenSCs isolation and culture

Isolation and culture of MenSCs were carried out according to the protocol we published elsewhere^55^ with minor modifications. In brief, menstrual blood was collected from volunteer women using Diva cup (Lunette, Sweden) on day 2 of the menstrual cycle, transferred into medium containing DMEM/F12 (Gibco, USA), fungizone, penicillin–streptomycin (all from Sigma, USA), and immediately transported to the laboratory in cold chain. Samples were washed and cultured in DMEM/F12 containing penicillin–streptomycin and 10% fetal bovine serum (FBS) (Gibco). Non-adherent cells were removed after 48 hrs, adhered MenSCs were detached using trypsin-EDTA (Gibco), and frozen for future experiments. BMSCs were cultured in parallel, as with MenSCs. All experiments were performed on MenSCs and BMSCs at passages 3–5. Isolated MSCs were characterized by immunophenotyping and multi-lineage differentiation as described in the following section.

### Characterization of MenSCs and BMSCs

Immunophenotyping of MSCs from both sources was carried out by flow cytometry using a panel of antibodies (Table 1). All immunostainings were done by direct staining with fluorochrome-labeled antibodies except for Oct-4. Intracellular staining of Oct-4 was performed on cells permeabilized with transcription factor buffer set (BD Pharmingen, USA). Cells were treated with rabbit anti-human Oct-4 antibody followed by incubation with FITC-conjugated goat anti-rabbit Ig (Abcam, UK). Signals were analyzed using Attune NxT flow cytometer (Thermo Fisher Scientific, USA) considering appropriate isotype controls.

**Table 1:** Antibodies.

Antibody	Fluorochrome	Clone	Manufacturer	Cat#
Anti-human CD9	FITC	M-L13	BD Pharmingen	555371
Anti-human CD10	PE	HI10a	BD Pharmingen	340921
Anti-human CD16	FITC	DJ130c	Dako	F7011
Anti-human CD29	PE	MAR4	BD Pharmingen	561795
Anti-human CD33	FITC	HIM3–4	BD Pharmingen	561818
Anti-human CD34	FITC	581	BD Pharmingen	560942
Anti-human CD38	FITC	HIT2	BD Pharmingen	560982
Anti-human CD44	PE	515	BD Pharmingen	550989
Anti-human CD45	PE	HI30	BD Pharmingen	560975
Anti-human CD56	APC	NCAM	BD Pharmingen	318310
Anti-human CD73	PE	AD2	BD Pharmingen	561014
Anti-human CD105	PE	166707	R&D systems	FAB10971P-025
Anti-human CD133	PE	W6B3C1	BD Pharmingen	566594
Anti-human CD107a	PE	H4A3	BD Pharmingen	555801
Anti-human granzyme A	PE	CB9	Biolegend	507206
Anti-human granzyme B	PE	GB11	BD Pharmingen	561142
Anti-Oct-4	—	Polyclonal	Abcam	ab19857
Anti-SSEA-4	PE	MC813-70	BD Pharmingen	560128
Anti-human IFN-γ	FITC	4 S.B3	Biolegend	502506
Anti-human IL-10	—	25209	R&D systems	MAB2171
Anti-human IL-6	—	P05231	R&D systems	MAB2061
Anti-human TGF-β	—	1D11	R&D systems	MAB1835
Mouse IgG1, κ Isotype Control	APC	—	BD Pharmingen	555751
Mouse IgG1, κ Isotype Control	PE	—	Biolegend	400111
Mouse IgG1, κ Isotype Control	FITC	—	BD Pharmingen	554679
Perforin Reagent Set	PE	δG9	BD Pharmingen	556437

To assess multi-lineage differentiation potential of the isolated MenSCs and BMSCs they were differentiated toward osteocytes, chondrocytes, and adipocytes using standard protocols^24,26,55^ and the extent of differentiation was then tested by Alizarin Red, Alcian Blue, and Oil Red O staining, respectively.

### NK cell purification and MenSCs-NK co-culture

NK cells were purified from buffy coat samples obtained from Iran Blood Transfusion Center (IBTC). In brief, mononuclear cells were isolated using Ficoll-Hypaque density gradient centrifugation (GE Healthcare, Sweden), and NK cells were negatively purified using NK cell isolation kit (Miltenyi Biotec, Germany) according to the manufacturer’s instruction. The purity of CD56 + NK cells was confirmed by flow cytometry. Optimal culture period and IL-2 concentration (R&D systems, USA) for NK cell recovery, viability, and proliferation were determined by cell counting, trypan blue staining (Gibco), and flow cytometry, respectively. Freshly isolated NK cells were cultured in 96-well plates containing RPMI-1640 (Gibco) supplemented with penicillin-streptomycin (100 µg/mL), L-glutamine (2 mM) (all from Sigma), non-essential amino acids, sodium pyruvate, and 10% FBS (all from Gibco) in the presence of different concentrations (100–800 U/mL) of recombinant human IL-2 for 4–6 days. Based on the results, concentration of 100 U/mL IL-2 for 5 days was selected as the optimal NK cell culture condition, based on which all subsequent experiments were performed. For the establishment of MenSCs-NK co-culture, freshly isolated NK cells were cultured in either the absence or presence of MenSCs at different ratios. Half of the medium was replaced with the freshly IL-2-supplemented medium on day 3 of co-culture which was continued for 2 further days. NK cells were collected on day 5 by gentle aspiration and analyzed for functional features. In functional studies listed below, BMSCs were also co-cultured with NK cells (1:5 and 1:10) (BMSCs:NK).

### Proliferation assay

The effect of MenSCs on NK cell proliferation was assessed in three different settings. In the first setting, purified NK cells were co-cultured in the absence or presence of optimally mitomycin C (Sigma)-inactivated, 25 µg/mL for 1 hrs, MenSCs (25 × 10^3^ MenSCs/well) at different ratios of MenSCs:NK (1:2-1:128) in a 96-well culture plate by using direct co-culture system for 5 days. NK cells were also co-cultured at the above ratios with MenSCs using the transwell 24-well plate (Corning, USA). In the second setting, MenSCs were pretreated with IFN-γ (25 ng/mL) and/or IL-1β (10 ng/mL) (all from PeproTech EC, UK) for 48 hrs and co-cultured with MenSCs with or without mitomycin C inactivation. In the third setting and to evaluate possible involvement of IL-6, IL-10, and TGF-β in MenSCs-modulated NK cell proliferation, IFN-γ/ IL-1β-pretreated MenSCs were co-cultured with NK cells in both presence and absence of IL-6, IL-10, and TGF-β neutralizing antibodies (10 µg/mL) (all from R&D systems). In all settings, NK cells were labeled with 5 µM 5,6-carboxyfluorescein diacetate succinimidyl ester (CFSE) (Molecular Probes, USA) before co-culture with MenSCs. Cultures were continued for 5 days, after which NK cells were harvested, stained with propidium iodide (PI) (Sigma), and proliferation was assessed by flow cytometry.

### Growth factor membrane Array

Growth factor profile of culture supernatants of MenSCs and BMSCs were analyzed by semi-quantitative Western spot blot analyses using human growth factor membrane antibody array (ab134002, Abcam) according to the manufacturer’s instruction. Both MSCs were cultured in DMEM/F12 in 24-well plates at a density of 10^5^ cells/well for 48 hrs. Then the supernatants were harvested, clarified by centrifugation and frozen at −80 °C until use. For quantification of growth factors, membranes were sequentially incubated with supernatants (overnight at 4 °C), biotin-conjugated anti-growth factor antibodies, HRP-conjugated streptavidin, and ECL detection system. Signals were captured at different time intervals by Fusion Fx image acquisition system (Vilber Lourmat, Torcy, France). Data were quantified via densitometry using the AlphaEaseFC software V3.1.2 with normalization to positive and negative controls. This process of normalization created a relative density value.

### Assessment of the IDO activity

MenSCs and BMSCs cells were stimulated with 100 ng/mL of IFN-γ for 48 hrs in complete phenol red-free DMEM/F12 medium (Sigma) in the presence of 100 µg/mL L-tryptophan (Sigma). The activity of IDO enzyme was measured by photometric determination of the kynurenine concentration in the supernatant as previously reported^56^. Briefly, 160 µL cell supernatant was transferred to a 96-well culture plate followed by addition of 10 µL of 30% trichloroacetic acid (TCA), for 30 min at 50 °C. 100 µL supernatant was then recovered after plate centrifugation at 400 g for 10 minutes and mixed with 100 µL freshly-prepared Ehrlich’s solution. Absorbance was read by Power Wave HT microplate spectrophotometer (Bio-Tek, USA) at 450 nm.

### NK cell cytotoxicity assay

Cytotoxicity of NK cells was measured separately in two settings against K562 cells and MenSCs as target cells using calcein-AM (cAM) fluorimetric assay (BD Pharmingen). For K562 cytotoxicity, freshly isolated NK cells were cultured in phenol red-free DMEM/F12 medium (Sigma) supplemented with 100 U/mL IL-2 either alone or in the presence of MenSCs at a ratio of 1:4 (MenSCs:NK). After 5 days, NK cells were collected and mixed with 2 mM cAM-loaded K562 cells (10^5^ cells) as target cells at a variable effector:target (E:T) ratios of 4:1, 2:1 and 1:1 in a final volume of 200 µL in 96-well U bottom plates (SPL, Korea). Notably, NK cells did not contain any major contamination of MenSCs (growing as adherent cells) as judged by CD56 + NK cells using flow cytometry. Each E:T ratio was tested in triplicate. After mixing, cell suspensions were incubated at 37 °C in 5% CO2 for 4 hrs. The spontaneous release of cAM was quantified by incubating cAM-loaded K562 in medium alone, and wells containing loaded target cells lysed by adding 2% Triton X-100 for 10 minutes served as maximum release control. Following 4 hrs incubation, plates were centrifuged at 400 g for 10 minutes, and 100 µL supernatant from each well was collected and transferred to a 96-well flat clear bottom, black wall plate (Corning) and the fluorescence signal was measured by FLx800 microplate fluorescence reader (Bio-Tek) at an excitation wavelength of 485 nm and emission wavelength of 530 nm. Cytotoxicity, measured as specific release percent of cAM, was calculated using the following equation:

Percent Specific Release = (Experimental release - Spontaneous release) /(Maximum release - Spontaneous release) × 100

To evaluate NK cell cytotoxicity effect on MenSCs, autologous and allogeneic MenSCs were used as targets. Briefly, MenSCs were plated at 25 × 10^3^ cells/well in a 96-well flat clear bottom, black wall plate. Co-culture with NK cells was performed at a 1:4 ratio (MenSCs:NK) for 24, 48 and 72 hrs. NK cells and the supernatant were then completely removed from the wells, adherent MenSCs were then loaded with 2 mM cAM for 30 minutes, and fluorescence intensity was measured by the microplate fluorescence reader. The negative control wells contained loaded target cells in the medium alone, and positive wells contained loaded target cells lysed by adding 2% Triton X-100. Corrected means (cm) were calculated by subtracting the mean fluorescent signal of positive control wells from those of either co-cultured (co-culture cm) or negative control wells (control cm). Percent of cytotoxicity was then calculated using the following equation:

Percent Specific Release = 100 - (Co-culture cm / Control cm) × 100

### Analysis of degranulation and cytokine release

To evaluate the MenSCs modulatory action on NK cell cytokines and cytotoxic proteins production, NK cells were harvested after 5 days co-culturing with MenSCs and were subsequently co-cultured with the K562 at a 1:1 ratio in U bottom 96-well culture plates (SPL) in the presence of 2 mM Golgi-stop (BD Pharmingen). After 4 hrs, cells were harvested and stained with the Live/Dead Fixable Near-IR (Life Technologies, Carlsbad, CA, USA) and APC-labeled anti-CD56. Cells were then fixed and permeabilized using transcription factor buffer set (BD Pharmingen) followed by staining using PE-labeled monoclonal antibodies against IFN-γ, perforin, granzymes A and B. In the case of CD107a expression, PE-labeled anti-CD107a was added to the wells of NK-K562 co-culture for 4 hrs. After that, cells were harvested and stained by Live/Dead Fixable Near-IR and APC-labeled anti-CD56 followed by flow cytometry analysis. In addition, CD16 expression on NK cells following 5 days co-culturing with MenSCs was also tested using FITC-labeled anti-CD16 by flow cytometry.

### *In vitro* decidualization

Decidualization of MenSCs was induced in phenol red-free DMEM-F12 supplemented with 2% charcoal-stripped FBS (Gibco) and penicillin–streptomycin by 5 days treatment with 0.5 mM 8-Bromoadenosine 3′,5′-cyclic monophosphate (8-Br-cAMP) and 1 µM medroxyprogesterone 17-acetate (MPA) (all from Sigma). Control culture plates received the same culture mediun as decidulaized cells except 8-Br-cAMP and MPA. In some settings, 25 ng/mL IFN-γ was added in the last 48 hrs of decidualization process. The expression of prolactin (*PRL*) and *IGFBP*-1 transcripts in decidualized and control cells was assessed by quantitative real-time PCR (qPCR) experiments using the primer set and the protocol published elsewhere^57^.

### Statistical analysis

All the statistical analyses and group comparisons were performed using GraphPad Prism 6. Data were compared with either Kruskal-Wallis or Mann-Whitney tests. Descriptive data were reported as mean ± standard error of the mean (SEM). P values less than 0.05 were interpreted as statistically significant. P values less than 0.05, 0.01, 0.001, and 0.0001 were shown as *, **, ***, and ****, respectively.

## References

[1] Manaster I (2008). Endometrial NK cells are special immature cells that await pregnancy. The Journal of Immunology.

[2] Moffett-King A (2002). Natural killer cells and pregnancy. Nature Reviews Immunology.

[3] Vacca P (2011). CD34+ hematopoietic precursors are present in human decidua and differentiate into natural killer cells upon interaction with stromal cells. Proceedings of the National Academy of Sciences.

[4] Carlino C (2008). Recruitment of circulating NK cells through decidual tissues: a possible mechanism controlling NK cell accumulation in the uterus during early pregnancy. Blood.

[5] Male V (2010). Immature NK cells, capable of producing IL-22, are present in human uterine mucosa. The Journal of Immunology.

[6] Hanna J (2003). CXCL12 expression by invasive trophoblasts induces the specific migration of CD16–human natural killer cells. Blood.

[7] Hanna J (2006). Decidual NK cells regulate key developmental processes at the human fetal-maternal interface. Nature medicine.

[8] Kalkunte SS (2009). Vascular endothelial growth factor C facilitates immune tolerance and endovascular activity of human uterine NK cells at the maternal-fetal interface. The Journal of Immunology.

[9] Koopman LA (2003). Human decidual natural killer cells are a unique NK cell subset with immunomodulatory potential. Journal of Experimental Medicine.

[10] Manaster I, Mandelboim O (2008). The unique properties of human NK cells in the uterine mucosa. Placenta.

[11] Hosseini S (2014). Comparative analysis of NK cell subsets in menstrual and peripheral blood of patients with unexplained recurrent spontaneous abortion and fertile subjects. Journal of reproductive immunology.

[12] Xu X (2012). Monocyte chemoattractant protein-1 secreted by decidual stromal cells inhibits NK cells cytotoxicity by up-regulating expression of SOCS3. PLoS One.

[13] Đokić J, Tomić S, Marković M, Milosavljević P, Čolić M (2013). Mesenchymal stem cells from periapical lesions modulate differentiation and functional properties of monocyte‐derived dendritic cells. European journal of immunology.

[14] Di Nicola M (2002). Human bone marrow stromal cells suppress T-lymphocyte proliferation induced by cellular or nonspecific mitogenic stimuli. Blood.

[15] Spaggiari GM (2008). Mesenchymal stem cells inhibit natural killer–cell proliferation, cytotoxicity, and cytokine production: role of indoleamine 2, 3-dioxygenase and prostaglandin E2. Blood.

[16] Spaggiari GM, Abdelrazik H, Becchetti F, Moretta L (2009). MSCs inhibit monocyte-derived DC maturation and function by selectively interfering with the generation of immature DCs: central role of MSC-derived prostaglandin E2. Blood.

[17] Liotta F (2008). Toll‐like receptors 3 and 4 are expressed by human bone marrow‐derived mesenchymal stem cells and can inhibit their T‐cell modulatory activity by impairing Notch signaling. Stem cells.

[18] Amsen D (2004). Instruction of distinct CD4 T helper cell fates by different notch ligands on antigen-presenting cells. Cell.

[19] Manaster I (2010). Notch activation enhances IFNγ secretion by human peripheral blood and decidual NK cells. Journal of reproductive immunology.

[20] Darzi S (2012). Osteogenic differentiation of stem cells derived from menstrual blood versus bone marrow in the presence of human platelet releasate. Tissue Engineering Part A.

[21] Kazemnejad S (2012). Characterization and chondrogenic differentiation of menstrual blood-derived stem cells on a nanofibrous scaffold. The International journal of artificial organs.

[22] Khanjani S (2014). Comparative evaluation of differentiation potential of menstrual blood-versus bone marrow-derived stem cells into hepatocyte-like cells. PLoS One.

[23] Khanmohammadi M (2012). Proliferation and chondrogenic differentiation potential of menstrual blood-and bone marrow-derived stem cells in two-dimensional culture. International journal of hematology.

[24] Khanmohammadi M (2014). Modified protocol for improvement of differentiation potential of menstrual blood‐derived stem cells into adipogenic lineage. Cell proliferation.

[25] Azedi F (2014). Differentiation potential of menstrual blood‐versus bone marrow‐stem cells into glial‐like cells. Cell biology international.

[26] Kazemnejad, S., Zarnani, A.-H., Khanmohammadi, M. & Mobini, S. in *Stem Cell**Nanotechnology* 149–169 (Springer, 2013).

[27] DelaRosa O (2011). Human adipose-derived stem cells impair natural killer cell function and exhibit low susceptibility to natural killer-mediated lysis. Stem cells and development.

[28] Puissant B (2005). Immunomodulatory effect of human adipose tissue‐derived adult stem cells: comparison with bone marrow mesenchymal stem cells. British journal of haematology.

[29] DelaRosa O (2009). Requirement of IFN-γ–mediated indoleamine 2, 3-dioxygenase expression in the modulation of lymphocyte proliferation by human adipose–derived stem cells. Tissue Engineering Part A.

[30] Nikoo S (2012). Effect of menstrual blood‐derived stromal stem cells on proliferative capacity of peripheral blood mononuclear cells in allogeneic mixed lymphocyte reaction. Journal of Obstetrics and Gynaecology Research.

[31] Bozorgmehr M (2014). Menstrual blood-derived stromal stem cells inhibit optimal generation and maturation of human monocyte-derived dendritic cells. Immunology letters.

[32] Krampera M (2006). Role for interferon‐γ in the immunomodulatory activity of human bone marrow mesenchymal stem cells. Stem cells.

[33] Vacca P (2010). Crosstalk between decidual NK and CD14+ myelomonocytic cells results in induction of Tregs and immunosuppression. Proceedings of the National Academy of Sciences.

[34] Dosiou C, Giudice LC (2004). Natural killer cells in pregnancy and recurrent pregnancy loss: endocrine and immunologic perspectives. Endocrine reviews.

[35] Croxatto D (2014). Stromal cells from human decidua exert a strong inhibitory effect on NK cell function and dendritic cell differentiation. PloS one.

[36] Giuliani M., Oudrhiri N., Noman Z. M., Vernochet A., Chouaib S., Azzarone B., Durrbach A., Bennaceur-Griscelli A. (2011). Human mesenchymal stem cells derived from induced pluripotent stem cells down-regulate NK-cell cytolytic machinery. Blood.

[37] Ni F (2013). IGF-1 promotes the development and cytotoxic activity of human NK cells. Nature communications.

[38] Chen J-Y, Mou X-Z, Du X-C, Xiang C (2015). Comparative analysis of biological characteristics of adult mesenchymal stem cells with different tissue origins. Asian Pacific journal of tropical medicine.

[39] Alcayaga-Miranda F (2015). Characterization of menstrual stem cells: angiogenic effect, migration and hematopoietic stem cell support in comparison with bone marrow mesenchymal stem cells. Stem cell research & therapy.

[40] Boissel L, Tuncer HH, Betancur M, Wolfberg A, Klingemann H (2008). Umbilical cord mesenchymal stem cells increase expansion of cord blood natural killer cells. Biology of Blood and Marrow Transplantation.

[41] Sotiropoulou PA, Perez SA, Gritzapis AD, Baxevanis CN, Papamichail M (2006). Interactions between human mesenchymal stem cells and natural killer cells. Stem cells.

[42] Aggarwal S, Pittenger MF (2005). Human mesenchymal stem cells modulate allogeneic immune cell responses. Blood.

[43] English K (2009). Cell contact, prostaglandin E2 and transforming growth factor beta 1 play non‐redundant roles in human mesenchymal stem cell induction of CD4+ CD25Highforkhead box P3+ regulatory T. cells. Clinical & Experimental Immunology.

[44] Luan X, Liu X (2010). Comparison the inhibitory effects of human bone marrow mesenchymal stem cells and human placenta mesenchymal stem cells on T cell proliferation. Xi bao yu fen zi mian yi xue za zhi=Chinese journal of cellular and molecular immunology.

[45] Djouad F (2010). Activin A expression regulates multipotency of mesenchymal progenitor cells. Stem cell research & therapy.

[46] Spaggiari GM, Capobianco A, Becchetti S, Mingari MC, Moretta L (2006). Mesenchymal stem cell-natural killer cell interactions: evidence that activated NK cells are capable of killing MSCs, whereas MSCs can inhibit IL-2-induced NK-cell proliferation. Blood.

[47] Michelo CM (2016). Added effects of dexamethasone and mesenchymal stem cells on early Natural Killer cell activation. Transplant immunology.

[48] Chatterjee D (2014). Role of gamma-secretase in human umbilical-cord derived mesenchymal stem cell mediated suppression of NK cell cytotoxicity. Cell Communication and Signaling.

[49] Zhong T (2018). Lectin histochemical analysis of uterine natural killer cells in normal, hydatidiform molar and invasive molar pregnancy. Oncology letters.

[50] Laban M, Ibrahim EA-S, Elsafty MSE, Hassanin AS (2014). Placenta accreta is associated with decreased decidual natural killer (dNK) cells population: a comparative pilot study. European Journal of Obstetrics & Gynecology and Reproductive Biology.

[51] Lucas, E., Dyer, N. P., Fishwick, K., Ott, S. & Brosens, J. Success after failure: the role of endometrial stem cells in recurrent miscarriage. *Reproduction*, REP-16–0306 (2016). 10.1530/REP-16-0306 27430234

[52] Casado JG, Tarazona R, Sanchez-Margallo F (2013). NK and MSCs crosstalk: the sense of immunomodulation and their sensitivity. Stem Cell Reviews and Reports.

[53] Wallace AE, Fraser R, Cartwright JE (2012). Extravillous trophoblast and decidual natural killer cells: a remodelling partnership. Human reproduction update.

[54] Helige C (2008). Trophoblastic invasion *in vitro* and *in vivo*: similarities and differences. Human reproduction.

[55] Kazemnejad S, Najafi R, Zarnani AH, Eghtesad S (2014). Comparative effect of human platelet derivatives on proliferation and osteogenic differentiation of menstrual blood-derived stem cells. Molecular biotechnology.

[56] Däubener W, Hucke C, Seidel K, Hadding U, MacKenzie CR (1999). Interleukin-1 inhibits gamma interferon-induced bacteriostasis in human uroepithelial cells. Infection and immunity.

[57] Huang J-Y, Yu P-H, Li Y-C, Kuo P-L (2017). NLRP7 contributes to *in vitro* decidualization of endometrial stromal cells. Reproductive Biology and Endocrinology.

